# Multi-objective spatial optimization of forest fire monitoring networks: An integrated GIS-MCDM framework enhanced by improved genetic algorithms

**DOI:** 10.1371/journal.pone.0338090

**Published:** 2025-12-19

**Authors:** Lijing Wang, Jike Feng, Jiayi Mao, Yadong Zhang, Junfan An

**Affiliations:** 1 College of Control and Mechanical Engineering, Tianjin Chengjian University, Tianjin, China; 2 College of Geoscience and Technology, Southwest Petroleum University, Chengdu, China; 3 Yangquan Municipal Natural Resources Satellite Application Center, Shanxi, China; 4 School of Environmental Science & Engineering, Tianjin University, Tianjin, China; Military University of Technology Faculty of Civil Engineering and Geodesy: Wojskowa Akademia Techniczna im Jaroslawa Dabrowskiego Wydzial Inzynierii Ladowej i Geodezji, POLAND

## Abstract

As one of the most destructive and rapidly spreading natural hazards, forest fires pose a severe threat to the stability of ecosystems. To effectively mitigate fire risks, this study proposes a site-selection model that integrates Multi-Criteria Decision Making (MCDM), Genetic Algorithm (GA), and Geographic Information System (GIS), with the aim of optimizing the spatial distribution of forest fire monitoring points and enhancing fire surveillance efficiency. The model is designed with three primary objectives: maximizing monitoring coverage, minimizing road network distance, and optimizing economic costs. To achieve adaptive decision-making, the Analytic Hierarchy Process (AHP) is employed to dynamically allocate objective weights. Building upon this, differential evolution operators and adaptive mechanisms are incorporated to strengthen the GA’s global search capability and convergence performance. Furthermore, GIS combined with the FUCOM method is utilized for suitability analysis of potential monitoring points, effectively excluding restricted zones such as lakes and farmland to ensure the rationality of site allocation. A case study conducted in a high fire-risk region of Shanxi Province, China, demonstrates that the improved GA exhibits superior performance in terms of convergence speed, solution quality, and stability. Moreover, the model enables flexible adjustment of objective weights according to decision-makers’ preferences, thereby generating multiple optimized site-selection schemes. Compared with conventional layouts, the optimized configuration achieves an 18.6% increase in monitoring coverage, along with reductions of 50% in point-to-road distance and 10.2% in economic costs. These findings highlight the effectiveness of the proposed model in multi-objective site selection optimization and provide robust, scientific decision support for the spatial planning of forest fire monitoring networks.

## 1. Introduction

Regarded as one of the most sudden, fast-spreading, and devastating natural disasters worldwide, forest fires pose a serious threat to the stability and security of ecological systems [1]. In recent years, their frequency has increased significantly, driven by the accelerated succession of forest ecosystems and the intensifying impacts of global climate change. According to the *Global Wildfire Statistics Report*, approximately 350 million hectares of forest are affected by wildfires each year, leading to substantial damage to the environment, human life and property, and critical infrastructure [2,3].

To effectively mitigate the risk of forest fires, many countries have accelerated the construction and improvement of forest fire monitoring systems [4,5], aiming to enhance early warning capacity and emergency response efficiency. A well-designed monitoring system enables the timely detection of fire dynamics, accurate assessment of fire scale and propagation trends, and supports decision-makers in formulating scientifically sound response strategies, thereby minimizing the adverse impacts of fires on society and the environment. Among the critical factors for ensuring the effectiveness of such systems, the spatial arrangement of monitoring points plays a pivotal role. Proper site selection not only guarantees comprehensive coverage of high-risk areas and improves the accuracy and timeliness of detection, but also reduces the likelihood of blind spots or redundant overlaps in surveillance. In contrast, inappropriate placement of monitoring points can result in inefficient resource utilization and delayed identification of early fire signals, ultimately hindering prompt emergency response. Consequently, optimizing the spatial allocation of monitoring points to maximize resource efficiency and system performance has become a pressing challenge that requires urgent attention.

Due to its powerful capabilities in storing, retrieving, interacting with, analyzing, and simulating real-world environmental data, the Geographic Information System (GIS) has become a widely used tool in site selection decision-making, particularly for managing and analyzing geospatial information [6]. For instance, L. Kareem [7] utilized GIS technology to identify optimal landfill sites. Chen [8] applied GIS to analyze optimal facility locations in the tourism industry. And Saleous [9] used GIS to determine favorable sites for wind farm construction. However, traditional GIS-based site selection methods primarily focus on the analysis of geographical spatial factors, relying on single-dimensional quantitative evaluation. Such approaches often lack the capacity to effectively integrate heterogeneous criteria from multiple sources, making it difficult to reconcile conflicting objectives and weight preferences, and thus fall short in addressing the demands of site selection under complex decision-making scenarios. The integration of GIS with Multi-Criteria Decision-Making (MCDM) techniques effectively overcomes these limitations, significantly enhancing the scientific rigor and accuracy of site selection decisions. For example, Elboshy [10] employed the Analytic Hierarchy Process (AHP) combined with GIS to conduct a comprehensive evaluation of criteria for photovoltaic system site selection in Egypt, generating a suitability map to guide system deployment. Manea [11] integrated the AHP method with the GIS ArcCatalog tool to strategically select airport locations in the Euphrates region. Similarly, Zhao [12] proposed a GIS-based site selection method combining fuzzy Decision-Making Trial and Evaluation Laboratory method (DEMATEL) and fuzzy Multi-Objective Optimization on the basis of Ratio Analysis (MULTIMOORA) to provide scientific support for photovoltaic charging station placement for electric vehicles in Qingdao. Although GIS–MCDM approaches can provide decision-makers with more precise and systematic analytical support, their reliance on static weight settings and linear aggregation strategies makes it difficult to capture the complex nonlinear interactions and interdependencies among multiple objectives. Consequently, when confronted with highly coupled, strongly conflicting, or dynamically evolving site selection problems, these methods still exhibit limitations in terms of flexibility and adaptability to changing environments. To address this, Zhang [13] proposed a monitoring site optimization model based on submodular function maximization to expand video surveillance coverage while reducing construction costs and time. Han [14] developed a location set covering model incorporating spatiotemporal data to optimize the layout of fire stations in Nanjing, significantly improving service coverage and reducing fire response times. Nevertheless, many of these site selection models are largely dependent on expert-defined parameters or incremental heuristic searches during the optimization process, which lack the capacity for global optimization and adaptive adjustment to conflicting objectives. As a result, when confronted with complex multi-objective requirements and dynamically changing conditions, such approaches thus remain limited in their ability to effectively capture multidimensional characteristics, nonlinear relationships, and the challenges posed by dynamic and uncertain environments.

With the continuous advancement of intelligent algorithms, the limitations of traditional optimization models in addressing complex decision-making problems have been effectively mitigated. Among them, machine learning methods, owing to their strong capacity to model complex nonlinear relationships between input and output variables [15], have been widely applied in diverse fields such as urban seismic vulnerability assessment [16], groundwater resource monitoring [17], and prediction of building material performance [18,19]. Meanwhile, metaheuristic algorithms, renowned for their superior global search ability and multi-objective optimization performance, have demonstrated remarkable adaptability and potential in solving complex spatial site selection problems characterized by multiple constraints and conflicting objectives. Consequently, they are gradually emerging as an indispensable technical approach in site selection decision-making research. For example, Bao [20] employed integer programming and a multi-objective genetic algorithm (GA) to develop a lookout tower site optimization model, significantly enhancing the efficiency of forest fire monitoring systems. Heyns [21] optimized the layout of camera tower sites using a multi-objective GA and a multi-resolution approach, improving deployment efficiency and monitoring coverage. Yang [22] proposed a drone deployment optimization method based on the Particle Swarm Optimization (PSO) algorithm, addressing monitoring and communication needs across diverse terrains and fire risks while enhancing efficiency and coverage. Bolouri [23] applied GA to solve the capacitated location-allocation problem for fire stations in District 11 of Tehran, while Masoumi [24] utilized the Non-dominated Sorting Genetic Algorithm-II (NSGA-II) to study industrial site selection in Zanjan Province, achieving significant results. Nagkoulis [25] used GA to identify optimal photovoltaic facility locations in La Palma del Condado, Spain, considering land use, environmental impact, and economic factors to minimize visual interference.

Despite their strong global search capability in multi-objective optimization and their ability to approximate the Pareto-optimal solution set, GA often suffer from slow convergence, susceptibility to premature local optima, and high sensitivity to parameter settings. Moreover, the absence of a well-balanced coordination mechanism between global exploration and local exploitation limits their effectiveness in addressing complex site selection problems. Consequently, numerous scholars have proposed targeted improvement strategies to enhance the stability and adaptability of GA. For example, Arns Steiner [26] improved GA adaptability and optimization capabilities through integer encoding and customized multi-objective strategies, achieving balanced healthcare micro-regionalization in Paraná, Brazil. Xie [27] proposed a directed crossover genetic algorithm with multi-level mutation mechanisms, significantly improving GA’s precision and convergence speed. Additionally, Gu [28] developed an adaptive maximum distance neighboring crossover strategy that dynamically adjusts the crossover probability based on population convergence, enhancing the algorithm’s adaptability and efficiency across different stages.

With the continuous advancement of site selection research, integrated models combining MCDM, GIS, and metaheuristic algorithms have attracted increasing attention due to their applicability and accuracy. Yang [29] proposed a decision model based on Grey Correlation Analysis (GCA), the Technique for Order Preference by Similarity to Ideal Solution (TOPSIS) and Improved Multi-Objective PSO for multi-objective scheduling in complex reservoir systems, including flood control, ecological protection, and water supply. By improving weight combination and ranking methods, the model effectively reconciles conflicting objectives and provides decision-makers with more advantageous support. Kaveh [30] developed a multi-objective site selection model that integrates AHP, GIS, and an Improved GA (IGA) to optimize the spatial layout of urban healthcare centers and hospital facilities. Beheshtifar [31] introduced a GIS-based multi-objective GA model for healthcare facility site selection, where the TOPSIS method was applied to evaluate optimal solutions under different weighting scenarios, thereby enhancing the rationality and adaptability of healthcare resource allocation in Tehran. Damos [32] proposed a GA-based urban tourism route optimization method that integrates GIS and AHP to improve both the efficiency and accuracy of route planning, and validated its effectiveness through a case study in Chengdu, China. Qasimi [33] employed GIS to analyze critical factors influencing wind farm siting, such as wind speed, topography, and climate, and combined AHP with GA to construct a multi-objective wind energy development model tailored to the geographical and climatic conditions of northern Afghanistan, providing reliable support for renewable energy planning. In summary, site selection optimization models integrating MCDM, GIS, and metaheuristic algorithms have been widely applied across diverse domains. However, systematic research specifically addressing the spatial allocation of forest fire monitoring points remains relatively scarce, highlighting the urgent need for further in-depth investigation.

In this paper, we propose an optimization model for the site selection of forest fire monitoring points that integrates MCDM, IGA, and GIS. The model aims to coordinate multiple conflicting objectives, including monitoring coverage, road network distance, and economic cost, in order to optimize the spatial layout of the monitoring network. To improve the model’s solving performance in complex multi-objective environments, differential evolution strategies and adaptive mechanisms are introduced to enhance the algorithm’s global search capability and convergence efficiency. Moreover, the model can dynamically adjust optimization strategies according to different preferences of decision-makers, generating more accurate and efficient site selection schemes under complex and variable terrain conditions, and providing flexible and reliable decision support for the scientific deployment of forest fire monitoring systems.

The structure of the paper is as follows: Section 2 introduces the site selection objectives and the construction of the multi-objective optimization model; Section 3 presents the improvements to the genetic algorithm; Section 4 conducts case validation; and Section 5 summarizes the findings and proposes recommendations for future research.

## 2. Materials and methods

### 2.1. Site selection model formulation

This study proposes a multi-objective optimization model combining MCDM and GIS to address the complex decision-making challenges in site selection for forest fire monitoring. First, site selection objective functions were constructed to identify the key factors involved in the decision-making process, with data analysis conducted using GIS technology. Next, the Weighted Sum Method (WSM) was employed to integrate multiple objectives into a comprehensive evaluation model, achieving a balance among different decision-making goals. To further accurately capture the preferences of decision-makers, the AHP was introduced, allowing for the effective expression of choices under conditions of uncertainty and subjective preferences. Finally, weights for the site selection objectives were calculated, providing a scientific and reliable quantitative foundation for subsequent optimization studies using IGA.

#### 2.1.1. Objective function.

Based on an in-depth investigation of site selection for forest fire monitoring, this study considers key factors such as monitoring effectiveness, accessibility, and cost-efficiency to construct three objective functions: monitoring coverage area, road network distance, and economic cost. Through quantitative analysis, the influence of each objective on site selection decisions is evaluated, providing a solid quantitative foundation for subsequent optimization and ensuring the rationality and scientific validity of the decisions.

(1) Monitoring coverage area

Monitoring coverage area is a key indicator for evaluating the effectiveness of a monitoring system, as it directly affects the timeliness and efficiency of fire warning. Therefore, this study optimizes the spatial distribution of monitoring points to maximize monitoring coverage, thereby enhancing the overall monitoring efficiency and emergency response capability of the system. The model is expressed as follows:


$$f_{1}=\sum_{i=1}^{N}\sum_{j=1}^{M}z_{ij}\cdot y_{i}$$ (1)

where N represents the total number of monitoring points, M represents the total number of demand points. *y*_*i*_ is a binary decision variable indicating whether monitoring point *i* is selected(*y*_*i*_ = 1) or not(*y*_*i*_ = 0). *z*_*ij*_ denotes the visibility between monitoring point *i* and demand point *j*. The higher the *f*_1_, the better the overall visibility.

**Fig 1:**
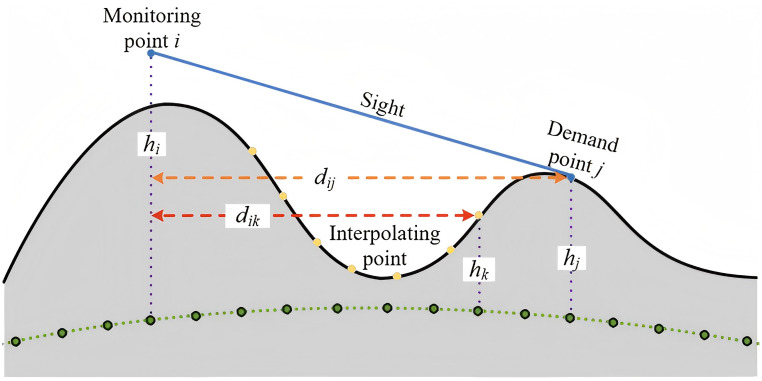
Visibility analysis between monitoring and demand points.

To determine whether a monitoring point *i* is visible from demand point *j*, the following condition is used:


$$z_{ij}=\left\{ \begin{array}{c} 1,\;\;ifh_{j}\leq h_{i}+\frac{h_{k}-h_{i}}{d_{ij}}\cdot d_{ik}\\ 0,\;otherwise \end{array}\right.$$ (2)

where, *h*_*i*_, *h*_*j*_ and *h*_*k*_ denote the elevations of monitoring point *i*, demand point *j*, and interpolation point *k*, respectively. *d*_*ij*_ and *d*_*ik*_ represent the horizontal distances between monitoring point *i* and demand point *j*, and between point *i* and interpolation point *k*, respectively. As shown in Fig 1, the interpolation point lies between the monitoring point and the demand point, and is used to determine whether the line of sight is obstructed by terrain. When the elevation values *h*_*k*_ of all interpolation points do not exceed the line-of-sight height (i.e., no obstruction occurs), *z*_*ij *_= 1; otherwise, *z*_*ij *_= 0.

This study constructed a Digital Elevation Model (DEM) based on a Triangulated Irregular Network (TIN) [34] to guarantee high-fidelity representation of terrain features, and employed GIS to extract the elevations of monitoring points, demand points, and interpolation points. The corresponding calculation relationships are as follows:


$$h_{j}=h_{DEM}(x_{j},y_{j}),h_{i}=h_{DEM}(x_{i},y_{i}),\;\;h_{k}=h_{DEM}(x_{k},y_{k})$$ (3)

where *h*_*DEM*_*(x,y)* denotes the elevation value of the DEM at coordinates *(x,y)*.

To ensure demand points are sufficiently monitored while considering visibility and distance limitations, the model introduces the following constraints:


$$\sum_{i=1}^{N}\sum_{j=1}^{M}z_{ij}\geq C_{j}$$ (4)

$$d_{ik}<d_{ij},\;\;d_{ij}\leq R_{m},\;\;\begin{array}{c} {}*20r\forall i,j \end{array}$$ (5)

where *C*_*j*_ is the minimum number of monitoring points required to cover demand points *j*, and *R*_*m*_ represents the maximum visible radius of monitoring point *i*.

(2) Road network distance

**Fig 2:**
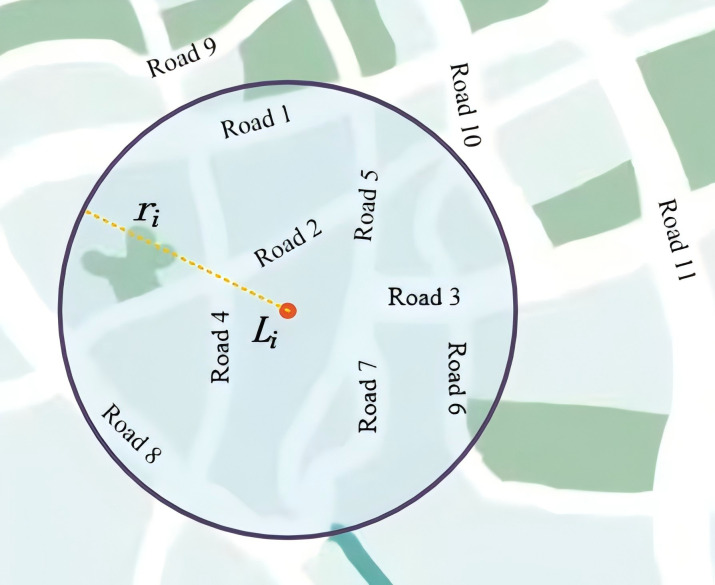
Spatial relationship of monitoring points and the road network.

In the site selection decision of forest fire monitoring systems, the distance between monitoring points and the road network is a critical factor influencing emergency response efficiency and operational convenience. Therefore, this study aimed to minimize the distance from monitoring points to the road network as an optimization objective, in order to facilitate rational spatial deployment, improve response speed, and reduce resource dispatching time. The model is expressed as follows:


$$f_{2}=\sum_{i=1}^{N}y_{i}\cdot d_{i}$$ (6)

where *d*_*i*_ represents the shortest distance between monitoring point *i* and the road network, and its calculation formula is as follows:


$$d_{i}=\underset{j\in J}{\min}(Ds(L_{i},R_{j},r_{i}))$$ (7)

where *L*_*i*_ and *R*_*j*_ represent the location of monitoring point *i* and road *j*, respectively, while set *J* comprises all roads. The function *Ds(L*_*i*_, *R*_*j*_, *r*_*i*_*)* is used to calculate the search radius *r*, defined as the straight-line distance between monitoring points and roads (Fig 2). To enhance computational efficiency and accuracy, the proximity analysis tool in GIS is employed for solution verification [35].

To ensure that the site selection scheme has reasonable road accessibility in spatial distribution, the following constraint is imposed:


$$\sum_{i=1}^{N}y_{i}\cdot d_{i}\leq D_{\max}$$ (8)

$$r_{i}\leq R_{m\mathrm{\backslash nolimits}},\;\;\begin{array}{c} {}*20r\forall i\in N \end{array}$$ (9)

where *D*_max_ represents the upper bound of the total shortest distance between monitoring points and roads.

(3) Economic cost

To ensure the economic feasibility of the site selection scheme, this study conducts a comprehensive evaluation of the construction and operation costs of monitoring points, with the objective of minimizing the total cost. Under the constraint of the budget, the spatial layout of monitoring points is optimized to achieve a coordinated balance between cost control and resource allocation efficiency. The model is expressed as follows:


$$f_{3}=\sum_{i=1}^{N}\left(C_{e}+C_{o}\right)\cdot y_{i}$$ (10)

where *C*_*e*_ represents the construction cost of monitoring points, and *C*_*o*_ represents the operation and maintenance cost of monitoring points, which are defined as follows:


$$C_{e}=c_{m}+c_{i}+k_{d}\cdot w\cdot d_{i}$$ (11)

$$C_{o}=\left(c_{r}+c_{n}\right)\cdot T+k_{p}\cdot d_{i}$$ (12)

where *C*_*e*_ includes the equipment installation cost *c*_*i*_, the equipment purchases cost *c*_*m*_, and the transportation cost. The transportation cost is related to the equipment weight *ω* and the distance *d*_*i*_ between monitoring points and roads, and is quantified by the transportation cost coefficient *k*_*d*_; *C*_*o*_ consists of the daily maintenance cost *c*_*r*_ and the communication network expense *c*_*n*_, which are annualized over the time horizon *T*. In addition, a maintenance path coefficient *k*_*p*_ is introduced to account for the additional cost associated with *d*_*i.*_

To ensure that the site selection scheme remains feasible within the budget, the following constraint is imposed:


$$\sum_{i=1}^{N}\left(C_{e}+C_{o}\right)\leq C_{\max}$$ (13)

where *C*_max_ denotes the maximum acceptable economic expenditure.

Furthermore, in the process of solving the multi-objective optimization problem, solutions that do not satisfy the above constraint are discarded in this study to improve computational efficiency, avoid infeasible schemes from entering subsequent calculations, and ensure that the optimization results meet practical deployment requirements.

#### 2.1.2. Multi-objective optimization model construction.

In the research on site selection for forest fire monitoring, the complexity of multi-objective optimization problems makes it challenging to evaluate and balance the trade-offs among objectives. Therefore, this study constructs a multi-objective optimization model to address the intricate conflicts of interest and the significant differences in priorities among the various objectives.

Firstly, a normalization method is employed to standardize objectives with different units and dimensions, thereby eliminating the influence of scale differences on the optimization process and ensuring effective trade-offs among objectives. The normalization formula is expressed as follows:


$${f_{u}}^{\prime }=\frac{f_{u}}{M_{u}}$$ (14)

where ${f_{u}}^{\prime }$ represents the normalized value of objective *u*, *f*_*u*_ denotes the actual value of the objective, and *M*_*u*_ is its standard quantization scale. In this study, *M*_*u*_ corresponds to the research area (268 square kilometers), the economic budget (CNY 25 million yuan), and the upper limit of distance (20 kilometers).

On this basis, the normalized objective values are integrated using a weighted sum method to construct a suitability function *F*, which is used for the comprehensive evaluation of multi-objective optimization. The mathematical expression is as follows:


$$F=\omega_{1}{f_{1}}^{\prime }+\omega_{2}\frac{\text{1}}{{f_{2}}^{\prime }}+\omega_{3}\frac{\text{1}}{{f_{3}}^{\prime }}$$ (15)

where *f*_*2*_ and *f*_*3*_ need to satisfy the minimization constraint conditions. Therefore, their normalized values ${f_{2}}^{\prime }$ and ${f_{3}}^{\prime }$ are inverted to achieve the maximization of the suitability function, facilitating decision-making analysis for site selection.

Next, to reflect the subjective preferences and requirements of decision-makers in the site selection process, this study adopts the AHP [36,37] to evaluate the importance of objectives. By constructing a pairwise comparison matrix, the relative importance between objectives is assessed, where the relative importance of each objective is quantified using a ratio scale (1–9). Based on these values, weights are assigned to each objective. To ensure the consistency of the judgment matrix, its maximum eigenvalue is calculated, and the consistency ratio (CR) test is conducted. When the CR value is less than 0.1, the judgment matrix is considered to have good consistency, and the weight allocation results are reasonable and reliable. The AHP effectively supports the decision-making process while enhancing the interpretability of the model.

Table 1 presents the levels of importance, with the judgment matrix element *a*_*ij*_ representing the relative importance of objective *i* to objective *j*, satisfying the following properties:

**Table 1:** The AHP method levels of importance.

Scale	Definition
1	Objective *i* and Objective *j* are equally important
3	Objective *i* is slightly more important than Objective *j*
5	Objective *i* is significantly more important than Objective *j*
7	Objective *i* is very much more important than Objective *j*
9	Objective *i* is extremely more important than Objective *j*
2,4,6,8	Objective *i* and Objective *j* are intermediate in importance


$$a_{ij}=\frac{\text{1}}{a_{ji}}$$ (16)

Subsequently, the geometric mean method is used to calculate the weights of the site selection objectives, and the maximum eigenvalue $\lambda_{\max}$ of the judgment matrix is determined. Based on this, the Consistency Index (CI) is calculated using the following formula:


$$CI=\frac{\lambda_{\max}-n}{n-1}$$ (17)

where *n* represents the order of the matrix.

Finally, to verify the rationality of the site selection method, a consistency test is conducted on the judgment matrix, and CR is calculated. The formula is as follows:


$$CR=\frac{CI}{RI}$$ (18)

where Random Index (RI) represents the random consistency index, the values of which can be obtained from Table 2. When CR < 0.1, the judgment matrix is considered to have good consistency, and the comparison results between the objectives are deemed reasonable and reliable. Otherwise, the matrix elements need to be adjusted until the consistency requirement is met. After passing the consistency test, the objective weights are incorporated into the construction of the fitness function to ensure the rationality and consistency of the evaluation criteria in the subsequent multi-objective optimization process.

**Table 2:** RI values for consistency verification.

n	1	2	3	4	5	6	7	8	9
RI	0	0	0.58	0.90	1.12	1.24	1.32	1.41	1.45

In summary, based on AHP, the importance of each site selection objective is quantified, and multiple weight combinations are determined according to different decision-making preferences, thereby constructing multiple fitness functions. On this basis, a genetic algorithm is introduced to optimize the site selection scheme, using the fitness functions as evaluation criteria to guide the search process toward station deployment schemes that gradually approach and better match the objective weight preferences. This, in turn, enhances the adaptability and practicality of the model in meeting multi-criteria decision-making requirements.

### 2.2. Improved genetic algorithm (IGA)

**Fig 3:**
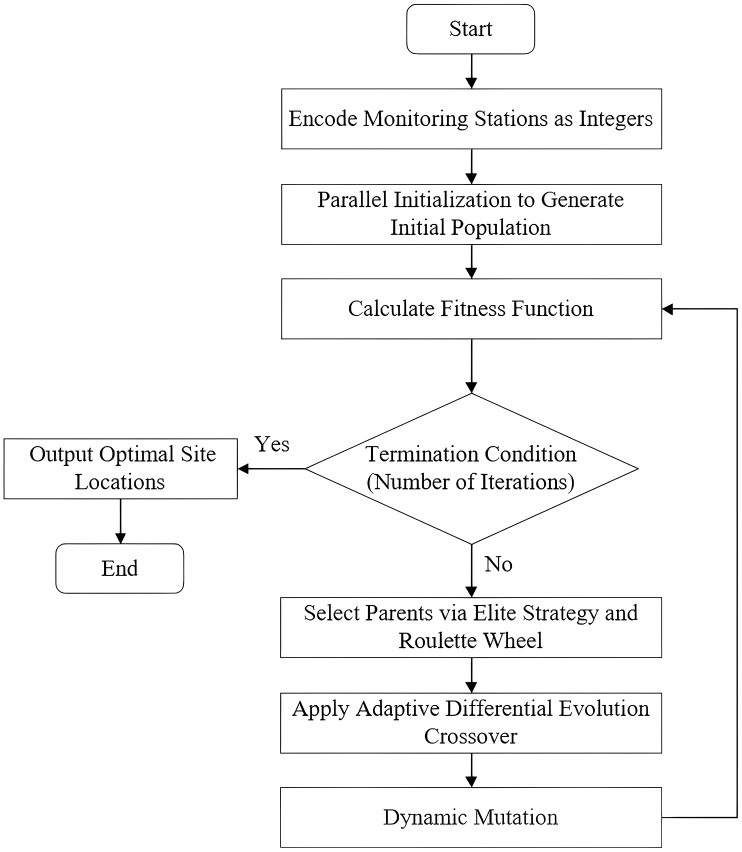
Flowchart of the IGA.

In this paper, considering that multiple objectives often involve significant conflicts and complex interrelationships, and that the site selection environment itself is characterized by high uncertainty and dynamic variability, traditional GA still face certain limitations in terms of search efficiency and convergence capability. To enhance the global search capability and computational efficiency of the algorithm, we introduce targeted improvements to the GA (Fig 3). Specifically, an initialization strategy is incorporated to boost computational efficiency; furthermore, adaptive mechanisms and differential evolution operators are combined with crossover and mutation operations to improve the stability and convergence speed of the search process.

#### 2.2.1. Encoding, parallel layered initialization, and fitness calculation.

To address the characteristics of site selection for forest fire monitoring points, this study adopts an integer encoding method, where the location of each monitoring point is represented by a unique identifier. This identifier serves as an index for monitoring point information. Such an encoding method effectively expresses the positional information of monitoring points and optimizes the crossover and mutation operations in the genetic algorithm, thereby accelerating the search for the global optimal solution.

Population initialization is a critical step in genetic algorithms, directly influencing the algorithm’s convergence accuracy and computational efficiency [38]. In this study, a number of points are randomly selected and combined to form initial solutions, with each solution representing a site selection scheme. To enhance computational performance and accommodate the demands of large-scale data processing, a parallel strategy is employed. Multiple threads collaborate to generate initial solutions, which are then merged into a complete population, ensuring population diversity while significantly improving initialization efficiency.

During fitness evaluation, the fitness values of solutions are calculated using Equation (15), providing a quantitative assessment of the quality of each site selection scheme and laying the foundation for subsequent genetic operations.

#### 2.2.2. Selection.

In the parent selection process, this study employs the roulette wheel selection method combined with an elitist strategy. This approach retains the high-quality individuals in the population while using random selection to effectively avoid getting trapped in local optima, thereby enhancing the global search capability and stability of the algorithm. The elite retention rate is set at 15%. For the remaining individuals, selection is performed using the roulette wheel method, as expressed by the following formula:


$$p(x_{i})=\frac{f(x_{i})}{\sum_{k=1}^{n}f(x_{k})}$$ (19)

where *p(x*_*i*_*)* represents the probability of individual *x*_*i*_ being selected, *f(x*_*i*_*)* denotes the fitness of the individual, *i* is the index of the individual, and *n* is the number of remaining individuals in the population.

#### 2.2.3. Improved crossover strategy.

GA typically use a fixed parent selection strategy, which limits the ability of the crossover operation to fully explore the solution space in the early stages. Additionally, they struggle to adjust the search direction dynamically based on the evolutionary dynamics of the population, resulting in slow convergence and insufficient precision in later stages. To address these issues, this study proposes a crossover strategy based on adaptive differential evolution. This strategy guides the search process using the positive and negative values of the differential vector, promoting convergence toward potential optimal solutions. It also dynamically adjusts the crossover operator according to the evolutionary state of the population to meet the search needs at different stages.

Step1: Adaptive Crossover Rate Adjustment

During the evolution process, the adaptive differential evolution algorithm dynamically adjusts the crossover rate *P*_*c*_ to ensure a relatively high crossover rate in the early stages of evolution, thereby increasing population diversity. In the later stages of evolution, the crossover rate is reduced to prevent excessive disturbance. The variation formula for the crossover rate *P*_*c*_ is as follows:


$$P_{c}=P_{\min}+(P_{\max}-P_{\min})\times \left(1-\frac{G_{c\mathrm{\backslash nolimits}}}{G_{\max}}\right)$$ (20)

where *P*_max_ and *P*_min_ represent the maximum and minimum crossover rates, respectively; *G*_*c*_ is the current generation number, and *G*_max_ is the maximum generation number.

Step 2: Generation of Differential Mutation Vector

The differential evolution algorithm is used to generate the mutation vector *V*_*i*_, and the formula for the mutation vector is as follows:


$$V_{i}=X_{best}+\beta \cdot (X_{r1}-X_{r2})$$ (21)

where *X*_*best*_ represents the individual with the best fitness in the current population, *X*_*r1*_ and *X*_*r2*_ are two random individuals from the population, and *β* is the scaling factor, typically taken within the range of [0, 1]. *r*1 and *r*2 are the index numbers of the individuals.

Step 3: Adaptive Differential Crossover

**Fig 4:**
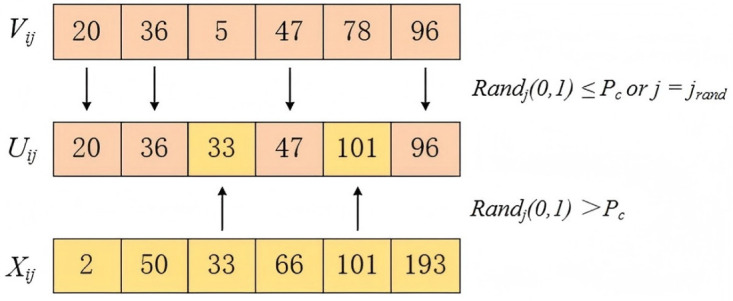
Differential evolution crossover process.

During the adaptive differential evolution crossover process, the trial vector *U*_*i*_ is generated through the crossover strategy, and its formula is as follows:


$$U_{i}=\{U_{i1},U_{i2},...,U_{iD}\}$$ (22)

where *D* represents the dimension of the chromosome. The determination method for each component *U*_*ij*_ in the trial vector is as follows:


$$U_{ij}=\left\{ \begin{array}{c} V_{ij}\;\;\;\;\;\;\;if\mathrm{\backslash nolimits}\begin{array}{c} {}*20rrand_{j}\left(0,\;1\right)\leq P_{c} \end{array}or\begin{array}{c} {}*20rj=j_{rand} \end{array}\\ X_{ij}\;\;\;\;\;\;otherwise\mathrm{\backslash nolimits} \end{array}\right.$$ (23)

where *V*_*ij*_ represents the *j*-th component value of the mutation vector *V*_*i*_, and *X*_*ij*_ denotes the *j*-th component of the current individual *X*_*i*_ in the population. As shown in Fig 4, when the condition $rand_{j}(0,1)\leq P_{c}$ or $j=j_{rand}$ is satisfied, *U*_*ij*_ is assigned the corresponding value of *V*_*ij*_; Otherwise, *U*_*ij*_ takes the corresponding value of *X*_*ij*_. Here, $rand_{j}(0,1)$ is a random variable uniformly distributed within the range [0, 1], and *j*_*rand*_ is an integer randomly selected from the range [1, D], ensuring that each trial vector includes at least one component derived from the mutation vector *V*_*i*_. This mechanism guarantees population diversity.

Step 4: Selection Based on Fitness Comparison

During the selection process, the trial vector *U*_*i*_ is compared with the current individual *X*_*i*_ based on their fitness values to determine whether the trial vector *U*_*i*_ will be accepted as a new chromosome for the next generation. The formula is as follows:


$${X_{i}}^{\left(t+1\right)}=\left\{ \begin{array}{c} U_{i}\;\;\;\;\;\;\;\;if\mathrm{\backslash nolimits}\;F\left(U_{i}\right)\;\;\geq \;\;F\left(X_{i}\right)\\ X_{i}\;\;\;\;\begin{array}{c} {}*20rotherwise \end{array} \end{array}\right.$$ (24)

where ${X_{i}}^{\left(t+1\right)}$ represents the individual in the next generation, *F(U*_*i*_*)* is the fitness value of the trial vector *U*_*i*_, and *F(X*_*i*_*)* is the fitness value of the current individual *X*_*i*_. Table 3 illustrates the detailed process of the improved crossover strategy.

**Table 3:** Differential evolution crossover process.

Algorithm1: Adaptive Differential Evolution Crossover Strategy
Input: Population size *N*, Maximum generations *G*_max._ *P*_max_, *P*_min_: Maximum and minimum crossover rates. *β*: Scaling factor in [0,1]. *D*: Dimension of chromosome. Output: Evolved population for next generation. 1. for *g* = 1 to *G*_max_ do 2. *P*_c _← *P*_min_ + (*P*_max_ - *P*_min_) × (1- *g*/ *G*_max_) ▷ Adaptive crossover rate 3. for each individual *X*_*i*_ in the population do 4. Select *X*_best_: best individual in current population 5. Randomly select two distinct individuals *X*_*r1*_, *X*_*r2*_ ≠ *X*_*i*_ 6. *V*_*i*_ ← *X*_best_ + *β*· (*X*_*r1*_ - *X*_*r2*_) ▷ Generate mutation vector 7. *j*_*rand*_ ← random integer in [1, *D*] 8. for *j* = 1 to *D* do 9. if rand (0,1) ≤ *P*_*c*_ or *j *== *j*_*rand*_ then 10. *U*_*ij*_ ← *V*_*ij*_ 11. else 12. *U*_*ij*_ ← *X*_*ij*_ 13. end if 14. end for 15. if *F(U*_*i*_) ≥ *F(X*_*i*_) then ▷*F*: fitness function 16. $X_{i}^{\left(next\right)}$ ← *U*_*i*_ 17. else 18. $X_{i}^{\left(next\right)}$ ← *X*_*i*_ 19. end if 20. end for 21. end for

#### 2.2.4. Dynamic mutation operator.

Traditional mutation operators typically use a fixed mutation rate, which cannot adjust dynamically based on individual fitness. This often leads to issues such as getting trapped in local optima or low search efficiency during the optimization process. To address this problem, this study introduces an adaptive mutation mechanism, where the mutation rate is dynamically adjusted according to the fitness of individuals. The formula is as follows:


$$P_{m}=\left\{ \begin{array}{c} K_{\min}+(K_{\max}-K_{\min})\cdot \frac{f_{\max}-f_{i}}{f_{\max}-\overset{―}{f}},\begin{array}{cc} {}*20c & f_{i}\geq \end{array}\overset{―}{f}\\ K_{\min}+(K_{\max}-K_{\min})\cdot \frac{f_{i}-f_{\min}}{\overset{―}{f}-f_{\min}},\begin{array}{cc} {}*20c & f_{i}<\overset{―}{f} \end{array} \end{array}\right.$$ (25)

where *K*_max_ and *K*_min_ represent the maximum and minimum mutation rates, respectively. *f*_*i*_ is the fitness of the individual, $\overset{―}{f}$ is the average fitness of the population, *f*_max_ is the fitness of the best individual in the population, and *f*_min_ is the fitness of the worst individual in the population. Table 4 illustrates the detailed process of the dynamic mutation operator.

**Table 4:** Calculation procedure for the adaptive mutation probability.

Algorithm2: Adaptive Mutation Probability Adjustment
Input: *f*_*i*_: Fitness value of the target individual. $\bar{f}$: Average fitness of the current population. *f*_max_: Maximum fitness (best individual) in the population. *f*_min_: Minimum fitness (worst individual) in the population. *K*_max_, *K*_min_: Maximum and minimum mutation rates. Output: *P*_*m*_: The adapted mutation probability for the target individual. 1. if *f*_*i*_ >$\bar{f}$ 2. ▷ For above-average individuals, inversely scale Pm based on proximity to best fitness 3. *P*_*m*_ ← *K*_min_ + (*K*_max_ - *K*_min_) × [(*f*_max_ – *f*_*i*_)/ (*f*_max_ -$\bar{f}$)] 4. else 5. ▷ For below-average individuals, scale Pm based on proximity to worst fit ness 6. *P*_*m*_ ← *K*_min_ + (*K*_max_ - *K*_min_) × [(*f*_*i*_ – *f*_min_)/ ($\bar{f}$- *f*_min_)] 7. end if 8. return *P*_*m*_

### 2.3. Study area

The study area is situated in the southwestern part of Shanxi Province, within the extended belt of the Lu’an Mountain Range (37°51’8’‘~38°3’50’‘N, 113°21’36’‘~113°35’47’‘E), encompassing approximately 268 km^2^. The regional geomorphology is primarily shaped by erosional mountainous features, with an overall topographic gradient descending from northwest (higher elevations) to southeast (lower elevations), and altitudes ranging from 700 to 1370 meters. Due to tectonic folding, the landscape has progressively developed comb-like ridges and deeply incised V-shaped valleys. The pronounced relief and complex spatial structure to some extent constrain the rational deployment and dispatch of video-based monitoring equipment.

The area exhibits distinct vertical vegetation zonation. At higher elevations, coniferous and mixed forests dominate, primarily composed of fir (*Abies* spp.) and Chinese pine (*Pinus tabuliformis*), both enriched in terpene-based volatile organic compounds. These forests are characterized by substantial litter accumulation and well-developed humus layers, rendering them highly prone to transition from smoldering to flaming combustion under extreme thermal conditions. In contrast, the lower-altitude hilly zones are extensively covered by deciduous broadleaf forests, mainly oak (*Quercus* spp.) and birch (*Betula* spp.), with dense Gramineae strata and intertwined climbing vegetation. Such undergrowth forms a vertically continuous fuel network, which accelerates fire spread and markedly elevates wildfire risk. Furthermore, the extensive vegetation cover, coupled with the spatial continuity of tall trees and dense shrubs, creates canopy layers that obstruct visibility and consequently impair the effectiveness of monitoring systems with respect to surveillance scope and emergency response capacity.

The area lies within a warm temperate semi-humid continental monsoon climate zone, characterized by distinct seasonal variations. The climate features hot and humid summers contrasted with cold and dry winters. Annual sunshine duration ranges between 2,700 and 2,900 hours, with total annual solar radiation reaching approximately 134 kcal/cm^2^. The mean annual temperature ranges from 8°C to 12°C, while annual precipitation varies between 450 and 550 mm, exhibiting clear seasonal patterns. During the dry season, the moisture content of forest fuels often drops below 12%, forming a “fuel accumulation window” conducive to fire outbreaks. Fire hazard levels are significantly higher during the months of May to July and October to November.

**Fig 5:**
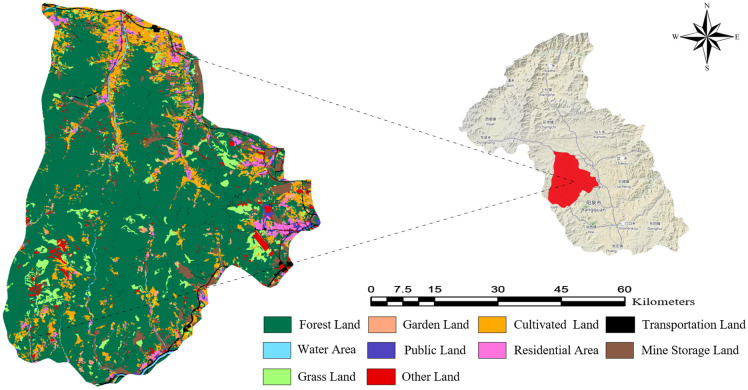
Categorization and spatial pattern of land-use types across the study area.

Therefore, this region was selected as the study area for optimizing wildfire monitoring point selection. Its complex topography, pronounced vegetation stratification, and seasonal wildfire risk provide challenging yet realistic conditions for validating the effectiveness of fire risk models. Fig 5 illustrates the distribution of land-use types within the region.

### 2.4. Data acquisition and processing

The data used in this study encompass a variety of geographic and environmental information to ensure the comprehensiveness and accuracy of the forest fire monitoring point selection analysis. The data include DEM, digital orthophoto map (DOM), land cover indices, solar radiation data, annual average temperature data, administrative boundary data, water system data, and village geographic information data.

**Fig 6:**
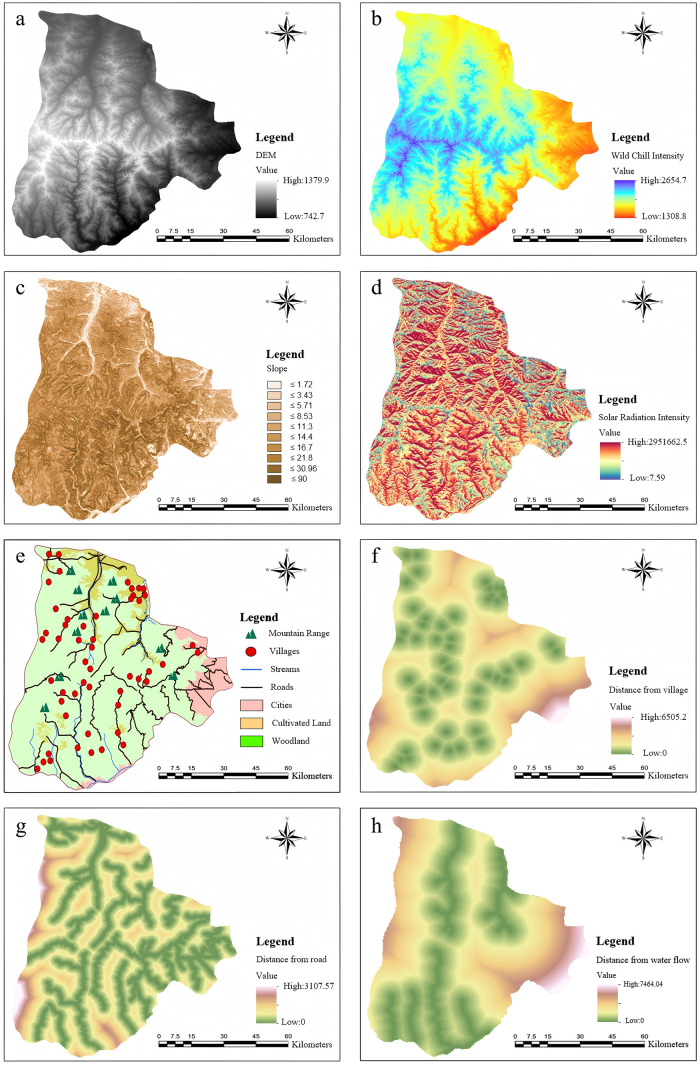
GIS-processed spatial data layers of site selection influencing factors. (a) DEM; (b) Wild chill intensity; (c) Slope; (d) Solar radiation intensity; (e) Vector data; (f) Distance from village; (g) Distance from road; (h) Distance from water flow.

To ensure the accuracy and consistency of the experiment, the data were normalized through a series of preprocessing steps. First, GIS was utilized to geocode the data and assign spatial attributes, addressing differences in format and units to enable precise distance calculations and spatial analysis. Second, the geocoded geographic information data were visually analyzed to intuitively present the spatial distribution characteristics of the study area. Finally, based on research requirements, lakes, streams, and agricultural lands were excluded from the monitoring point selection areas. The data processing results are shown in Fig 6.

After data preprocessing, in order to further improve the accuracy of wildfire monitoring point selection, eight evaluation indicators were considered: altitude, slope, land-cover type, solar radiation, mean annual temperature, wind speed, distance from rivers, and distance from roads. The Full Consistency Method (FUCOM) was employed to assign weights to these indicators [39,40].

FUCOM is a multi-criteria decision-making approach based on pairwise comparisons, designed to determine the relative importance of decision criteria. Experts or stakeholders first construct a judgment matrix to quantify the relative importance of each criterion on a 1–9 scale. The analysis then yields the final weights of the indicators. To ensure allocation consistency and rationality, the method introduces the Deviation from Full Consistency (DFC) index, which minimizes the deviation within the judgment matrix.

Considering the potential complexity and interrelationships among the evaluation indicators, the FUCOM is more suitable for this study than AHP. Moreover, FUCOM requires significantly fewer pairwise comparisons (FUCOM requires *n–1* comparisons, whereas AHP requires *n(n–1)/2* comparisons), which substantially reduces redundant comparisons, improves computational efficiency, and ensures more reliable results [41].

**Fig 7:**
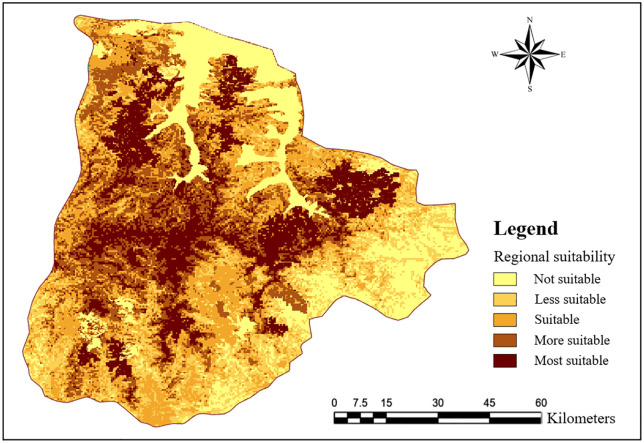
FUCOM-Based suitability map for forest fire monitoring point placement in the study area.

Based on the weights derived from FUCOM, the evaluation indices were integrated into a weighted overlay analysis to generate a suitability map for monitoring point selection. The suitability levels were classified into five categories: most suitable, suitable, moderately suitable, less suitable, and unsuitable (Fig 7). Subsequently, using the “Create” function in ArcGIS Pro, 200 optimal monitoring points were extracted by constraining different levels of suitability. This optimization process provides a robust scientific basis for wildfire monitoring point selection and offers practical data support for decision-making.

## 3. Results

To verify the effectiveness of the IGA, a case study on forest fire monitoring point selection was conducted, and its performance was compared with that of GA and PSO. To ensure the fairness of the comparison, equal weights were assigned to all objectives during the experimental process. The parameter configurations of IGA, GA and PSO were set according to the studies of Krajčovič [42] and Isiet [43], as detailed in Table 5. All experiments were implemented on a high-performance computing platform equipped with an Intel Core i7 processor, 16 GB RAM, and an NVIDIA GeForce GTX 1080 GPU to ensure computational efficiency.

**Table 5:** Parameter settings for the IGA, GA and PSO.

IGA	GA	PSO
Population size *P* = 200	Population size *P* = 200	Population size *P* = 200
Number of iterations *N* = 500	Number of iterations *N* = 500	Number of iterations *N* = 500
Minimum crossover rate 0.75 Maximum crossover rate 0.95	Crossover rate 0.95	Inertia weight *ω* = 0.5
Minimum mutate rate 0.05 Maximum mutate rate 0.15	Mutate rate 0.15	Cognitive coefficient *c*1 = 2.5
Step 100	Step 100	Social coefficient *c*2 = 1

**Fig 8:**
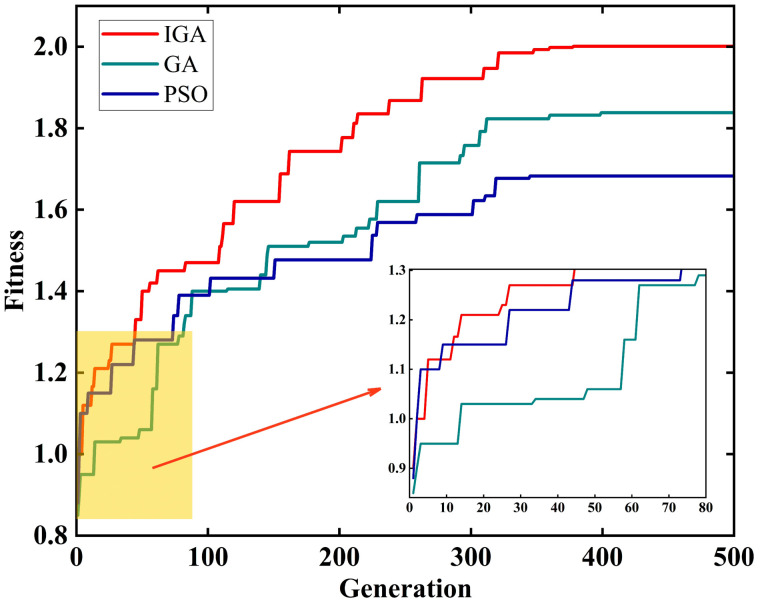
Comparison of fitness convergence curves for IGA, GA and PSO.

Fig 8 illustrates the comparative performance of the IGA, GA, and PSO in the multi-objective site selection optimization problem. It can be observed that the three algorithms exhibit significant differences during the evolutionary process.

During the early stage of evolution, IGA can quickly capture high-quality solutions, leading to a remarkable improvement in fitness values. GA also shows an upward trend, but its convergence speed is significantly slower. In the mid-stage, the convergence curve of GA tends to flatten, indicating stagnation in local optima and a decline in overall search efficiency. In contrast, IGA integrates a differential evolution operator with adaptive mechanisms, which enhances population diversity and broadens the search space, thereby maintaining relatively high search activity. In the late stage, the fitness convergence curve of IGA gradually stabilizes, demonstrating superior ability to avoid premature convergence and to identify globally optimal solutions, while GA shows limited overall performance.

Meanwhile, PSO demonstrates relatively strong global search ability during the early and mid stages of evolution, and its fitness values improve steadily. However, its particle updates heavily rely on individual and group best information. This mechanism makes PSO more suitable for continuous optimization problems, but in the discrete optimization problem investigated in this study, its weak ability to maintain population diversity leads to premature convergence in the mid and late stages, reducing its exploration capacity and making it prone to local optima.

In summary, under the same experimental conditions, IGA achieves better global search efficiency and robustness than GA and PSO, and can effectively balance convergence speed and solution stability, thereby demonstrating superior adaptability and stability in solving complex multi-objective optimization problems.

Although IGA progressively improves the overall fitness of the site selection objectives, inherent conflicts between objectives can cause the algorithm to prioritize the objectives with greater impact on overall fitness changes, forcing other objectives to make moderate compromises. This mechanism may result in solutions with high overall fitness values but suboptimal performance for certain individual objectives.

**Fig 9:**
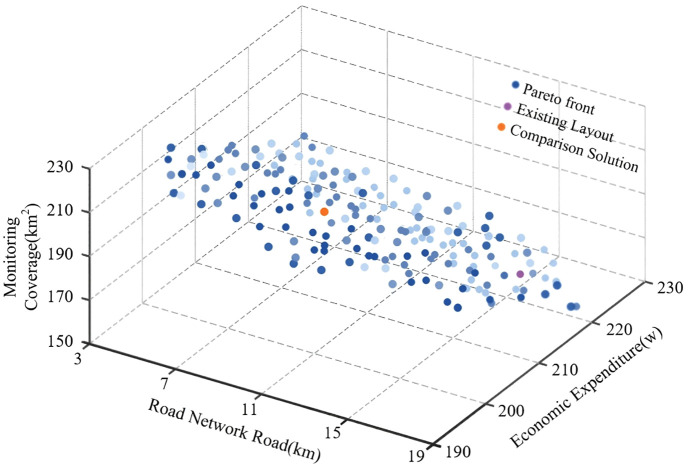
Evolutionary formation of the Pareto-optimal solution set for multi-objective monitoring point placement. (The varying shades of blue in the circular markers are used solely to indicate positional differences and have no additional significance).

To better understand the diversity of solutions and the balance among objectives during the optimization process, this study further analyzes the solution set generated by the algorithm using Pareto solutions based on the above optimization results. Although the multi-objective optimization was combined into a single fitness function during the computation, the independent values of each objective for each site selection result were retained. Therefore, a Pareto solution set was constructed using a non-dominated sorting approach. Fig 9 illustrates the evolutionary path of the algorithm during the search for optimal solutions, clearly demonstrating the dynamic trade-offs among monitoring coverage, road network distance, and economic cost. As the iterations progress, the solutions are gradually optimized, ultimately yielding multiple relatively balanced solutions.

To further validate the feasibility of the optimization results, a comparative analysis was conducted between a selected solution from the Pareto set and the existing layout. As presented in Table 6, the optimized site selection scheme demonstrated significant overall improvement: the monitoring coverage area increased from 1.538 × 10^8^ to 1.795 × 10^8^, significantly expanding the monitoring range; the distance between monitoring points and the road network decreased from 1.36 × 10^4^ to 1.064 × 10^4^ and the economic cost reduced from 2.198 × 10^6^ to 2.146 × 10^6^, reflecting better cost-effectiveness. These results indicate that the proposed optimization method performs well in multi-objective optimization and provides scientific evidence and practical solutions for the forest fire monitoring point selection problem.

**Table 6:** Comparison of site selection objectives.

Objective	Existing layout	Comparison solution
Monitoring coverage area (m^2^)	1.538 × 10^8^	1.795 × 10^8^
Road network distance (m)	1.36 × 10^4^	1.064 × 10^4^
Economic cost (CNY)	2.198 × 10^6^	2.146 × 10^6^

## 4. Discussion

### 4.1. Parameter sensitivity analysis

In the model application process, variations in parameter settings may lead to fluctuations in the results. Therefore, it is necessary to conduct sensitivity analysis to evaluate the stability and reliability of the model under parameter changes. In this study, the monitoring radius *R*_max_ was selected as a key parameter, as it not only determines the visible range of monitoring points but also largely affects the spatial distribution and coverage level of the entire monitoring network. If this parameter is improperly set, it may result in insufficient coverage or resource waste. Consequently, sensitivity analysis was performed on *R*_max_, with its values set to 3 km, 5 km, 7 km, 9 km, and 11 km under the condition that other parameters remained unchanged, in order to compare the variations in different indicators across scenarios (Table 7).

**Table 7:** Sensitivity analysis results under different monitoring radius.

Scenario	*R*_max_ (km)	Number of monitoring points	Monitoring coverage area (m^2^)	Road network Distance (m)	Economic cost (CNY)	Fitness value
1	3	20	1.323 × 10^8^	7.111 × 10^3^	2.011 × 10^6^	1.516
2	5	20	1.576 × 10^8^	7.983 × 10^3^	2.033 × 10^6^	1.441
3	7	20	1.829 × 10^8^	8.758 × 10^3^	2.085 × 10^6^	1.388
4	9	20	1.952 × 10^8^	1.015 × 10^4^	2.062 × 10^6^	1.304
5	11	20	2.011 × 10^8^	9.523 × 10^3^	2.111 × 10^6^	1.345

The experimental results indicate that with the increase of *R*_max_, the monitoring coverage area expands significantly, while variations in road network distance and economic costs remain relatively small. Moreover, the fitness function values remain stable, demonstrating that the model maintains good robustness under parameter adjustments. In practical site selection decisions, the setting of the monitoring radius should comprehensively consider factors such as terrain conditions, fire risk, and economic investment to ensure the scientific validity and operability of the scheme. Based on field survey statistics, *R*_max_ was set to 6 km in subsequent experiments to enhance the feasibility of the scheme.

### 4.2. Performance comparison of preference-based schemes

Decision-makers often assign different levels of importance to objectives based on varying interests and requirements in practical applications. To account for this, the present study further constructed multiple scenarios with different weight distributions of objectives, and the AHP was employed to quantify the weights of each objective. This allowed for the analysis of differences in site selection results under biased conditions. To ensure the logical consistency and computational reliability of the judgment matrices, a consistency check was conducted. As shown in Table 8, the CR of all scenarios were less than 0.1, indicating that the judgment matrices had satisfactory consistency and that the weight allocation results were both reasonable and reliable. It should be noted that if the CR value of a scenario exceeds 0.1, this suggests insufficient consistency in the judgment matrix, necessitating an adjustment of the relative importance among objectives to ensure the scientific rigor and reliability of the analysis results.

**Table 8:** AHP judgment matrices and weights for different preference schemes.

Scheme 1				
	**Monitoring coverage area**	**Road network distance**	**Economic cost**	**Weight**
Monitoring coverage area	1	3	5	0.627
Road network distance	1/3	1	4	0.28
Economic cost	1/5	1/4	1	0.093
The **CR = 0.072 < 0.1**, which confirms the consistency of the judgment matrix.				
Scheme 2				
	**Monitoring coverage area**	**Road network distance**	**Economic cost**	**Weight**
Monitoring coverage area	1	1/2	3	0.319
Road network distance	2	1	4	0.558
Economic cost	1/3	1/4	1	0.123
The **CR = 0.016 < 0.1**, which confirms the consistency of the judgment matrix.				
Scheme 3				
	**Monitoring coverage area**	**Road network distance**	**Economic cost**	**Weight**
Monitoring coverage area	1	1/2	1/5	0.128
Road network distance	2	1	1/2	0.276
Economic cost	5	2	1	0.596
The **CR = 0.004 < 0.1**, which confirms the consistency of the judgment matrix.				
Scheme 4				
	**Monitoring coverage area**	**Road network distance**	**Economic cost**	**Weight**
Monitoring coverage area	1	1	1	1/3
Road network distance	1	1	1	1/3
Economic cost	1	1	1	1/3
The **CR = 0 < 0.1**, which confirms the consistency of the judgment matrix.				

To evaluate the impact of different preference weights on site selection outcomes, this study optimized the schemes using IGA based on the weights described above. As shown in Table 9, each scheme demonstrates distinct advantages in terms of monitoring coverage, road network accessibility, and economic cost.

**Table 9:** Performance metrics of optimization schemes under different preferences.

Scheme	Weight	Monitoring coverage area (m^2^)	Road network Distance (m)	Economic cost (CNY)
1	[0.627, 0.28, 0.093]	2.034 × 10^8^	1.092 × 10^4^	2.181 × 10^6^
2	[0.319, 0.558, 0.123]	1.682 × 10^8^	3.6 × 10^3^	2.109 × 10^6^
3	[0.128, 0.276, 0.596]	1.545 × 10^8^	5.95 × 10^3^	1.944 × 10^6^
4	[1/3, 1/3, 1/3]	1.954 × 10^8^	9.73 × 10^3^	2.141 × 10^6^

**Fig 10:**
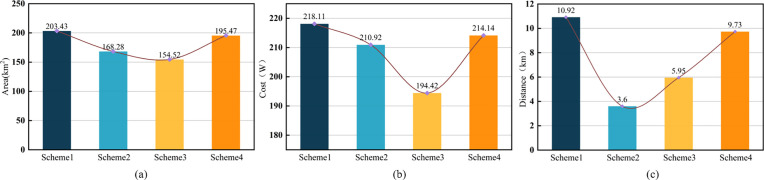
Comparison of key performance indicators across the four preference-based schemes. **(a)** Monitoring coverage; **(b)** Economic expenditure; **(c)** Road network distance.

In Scheme I, which prioritizes monitoring coverage, the total coverage increases from 1.538 × 10^8^ m^2^ to 2.034 × 10^8^ m^2^, representing an 18.6% improvement that significantly enhances wildfire perception and response capability. At the same time, the distance between monitoring points and the road network is moderately optimized, although economic costs control remains relatively constrained. Consequently, this scheme is more suitable for mountainous and hilly regions characterized by dense vegetation, high wildfire risk, and stringent requirements for monitoring accuracy and response efficiency. Scheme II places greater emphasis on the spatial coordination between monitoring points and the road network. After optimization, the average distance is reduced by 50% (from 1.36 × 10^4^ m to 3.6 × 10^3^ m), which markedly improves traffic accessibility. Meanwhile, by maintaining a certain level of monitoring coverage, this scheme achieves effective economic costs control, making it more applicable in contexts where efficient resource allocation and rapid traffic response are critical. Scheme III demonstrates a distinct advantage in economic costs control. Through rational spatial optimization, the total expenditure decreases from CNY 2.198 × 10^6^ to CNY 1.944 × 10^6^, a reduction of 10.2%. Although its monitoring coverage is relatively weaker, it is particularly appropriate where budget constraints are tight or cost-effectiveness is a primary concern. In contrast, Scheme IV, which balances multiple objectives, delivers the most well-rounded siting performance. Compared with the original layout, its monitoring coverage increases from 57.3% to 72.9%, while the average distance between monitoring points and the road network decreases from 1.36 × 10^4^ m to 9.73 × 10^3^ m. At the same time, economic costs are also reduced, enabling Scheme IV to achieve coordinated optimization of coverage capacity, traffic accessibility, and cost control (Fig 10), and making it especially suitable for applications that require comprehensive and balanced performance.

In summary, the site optimization model developed in this study is capable of comprehensively accounting for monitoring coverage, traffic accessibility, and economic costs, thereby providing decision-makers with targeted and adaptive siting solutions under different scenarios. In practical applications of forest fire monitoring point placement, where decisions are constrained by natural, social, and economic factors, decision-makers can set specific objectives and adjust weights according to actual needs to generate preferred solutions. Among these, the balanced optimization scheme obtained under a scenario with equal weighting of all objectives and no specific preference can serve as a reference baseline for further adjustments. When a particular objective is assigned higher priority in a specific context, its performance can be enhanced in a targeted manner based on this benchmark. Thereby, the decision-making process becomes supported by quantifiable data evaluations, shifting from goal-driven to evidence-based site selection, which better aligns with practical requirements and improves the scientific rigor and accuracy of decisions.

**Fig 11:**
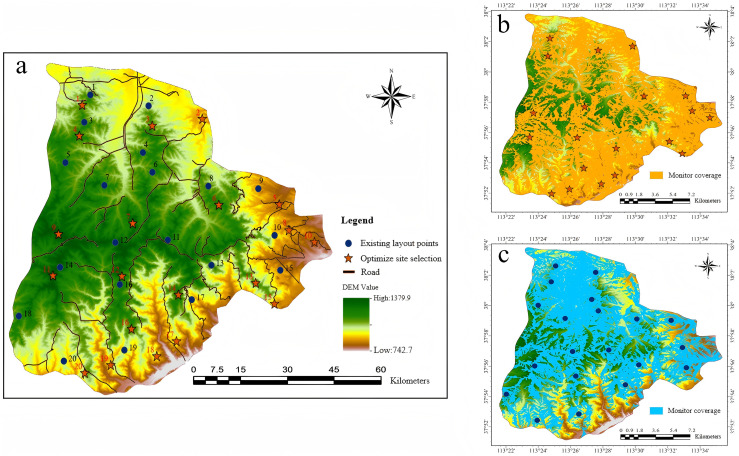
Spatial distribution and coverage comparison between the optimized and existing monitoring point layouts. (a) Comparison of monitoring points; (b) Monitoring coverage area of the optimized site selection scheme;(c) Monitoring coverage area of the existing layout.

To further evaluate the optimization outcomes, the proposed site selection plans were spatially visualized using GIS and compared with the existing layout of monitoring points. Fig 11 illustrates the spatial distribution and monitoring coverage of the balanced optimization scheme alongside the current configuration, clearly demonstrating the differences in spatial allocation and coverage capability before and after optimization. During the site selection analysis, decision-makers can use GIS to load model outputs and perform multi-dimensional visual comparisons by overlaying key factors such as road accessibility, vegetation coverage, and terrain conditions through layer control and zoom functions. Moreover, by incorporating an interactive analysis module, parameters such as monitoring radius and elevation can be dynamically adjusted, with visualization results updated in real time, facilitating the identification of the optimal solution under given preferences and constraints. Therefore, by leveraging GIS capabilities for multi-source data integration and spatial analysis, the intuitiveness and interpretability of the model’s optimized results are significantly enhanced. This approach also improves the transparency and operability of the decision-making process, offering robust technical support for the evaluation and selection of forest fire prevention and control strategies.

## 5. Conclusions

This paper proposes a site selection model that integrates MCDM, IGA, and GIS to optimize the spatial distribution of forest fire monitoring points. Experimental results demonstrate that the improved genetic algorithm exhibits strong global optimization capability, efficiently explores the solution space, and achieves an effective balance among multiple objectives including monitoring coverage area, road network distance, and economic cost. By introducing an objective weighting strategy, the model can generate flexible site selection plans adapted to different decision-making preferences. Specifically, a coverage-prioritized scheme increased the monitoring coverage area by 18.6%, proving particularly suitable for high-fire-risk regions. A scheme emphasizing road accessibility reduced the road network distance by 50%, making it more applicable to scenarios requiring rapid response. Meanwhile, an economically-focused scheme reduced costs by 10.2%, better suiting decision-making under resource constraints. Furthermore, the integration of GIS for visualizing the solutions not only enhanced the interpretability of the proposals but also facilitated intuitive comparison between different options, further validating the model’s flexibility and practical utility in addressing diverse demands. In summary, this study provides a reliable theoretical foundation and decision-making support for the scientific planning of forest fire monitoring points.

While this study has achieved certain results in addressing the optimization of forest fire monitoring point placement, several limitations remain. First, due to its high computational complexity, IGA exhibits slow global search performance when processing large-scale datasets. Subsequent research could integrate IGA with other meta-heuristic algorithms or machine learning methods to reduce runtime and enhance overall computational performance. Second, the model validation in this study was confined to a single high fire-risk region and has not been applied to areas with different fire characteristics. Future research should extend the model’s application to regions with diverse topography, socioeconomic conditions, climate types, and fire risk patterns. Corresponding optimization objectives and constraints should be established based on decision-making needs, with adaptive adjustments to model parameters and weight configurations to improve its stability and adaptability in varied environments. Furthermore, the model could be combined with technologies such as real-time remote sensing and Unmanned Aerial Vehicle (UAV)-based fire detection to enable rapid data acquisition and comprehensive analysis of environmental changes. These results could then be integrated into fire monitoring and early warning platforms to support rapid fire identification, assessment, and response. On this basis, by incorporating situational analysis and resource assessment, dynamic optimization of monitoring point deployment and response strategies can be achieved, thereby further enhancing forest fire monitoring and prevention capabilities.

## Supporting information

S1 Raw Data This is the original data1 required to replicate the manuscript’s results. (ZIP)

S2 Raw Data This is the original data2 required to replicate the manuscript’s results. (ZIP)

S3 Raw Data This is the original data3 required to replicate the manuscript’s results. (ZIP)

S4 Raw Data This is the original data4 required to replicate the manuscript’s results. (ZIP)

S5 Raw Data This is the original data5 required to replicate the manuscript’s results. (ZIP)

S6 Raw Data This is the original data6 required to replicate the manuscript’s results. (ZIP)

S7 Raw Data This is the original data7 required to replicate the manuscript’s results. (ZIP)

## Abbreviations

GIS: Geographic Information System
MCDM: Multi-Criteria Decision-Making
AHP: Analytic Hierarchy Process
DEMATEL: Decision-Making Trial and Evaluation Laboratory
MULTIMOORA: Multi-Objective Optimization on the basis of Ratio Analysis
GA: Genetic Algorithm
IGA: Improved Genetic Algorithm
PSO: Particle Swarm Optimization
NSGA-II: Non-dominated Sorting Genetic Algorithm-II
GCA: Grey Correlation Analysis
TOPSIS: Technique for Order Preference by Similarity to Ideal Solution
WSM: Weighted Sum Method
DEM: Digital Elevation Model
TIN: Triangulated Irregular Network
CR: Consistency Ratio
CI: Consistency Index
DOM: Digital Orthophoto Map
FUCOM: Full Consistency Method
DFC: Deviation from Full Consistency
UAV: Unmanned Aerial Vehicle.
